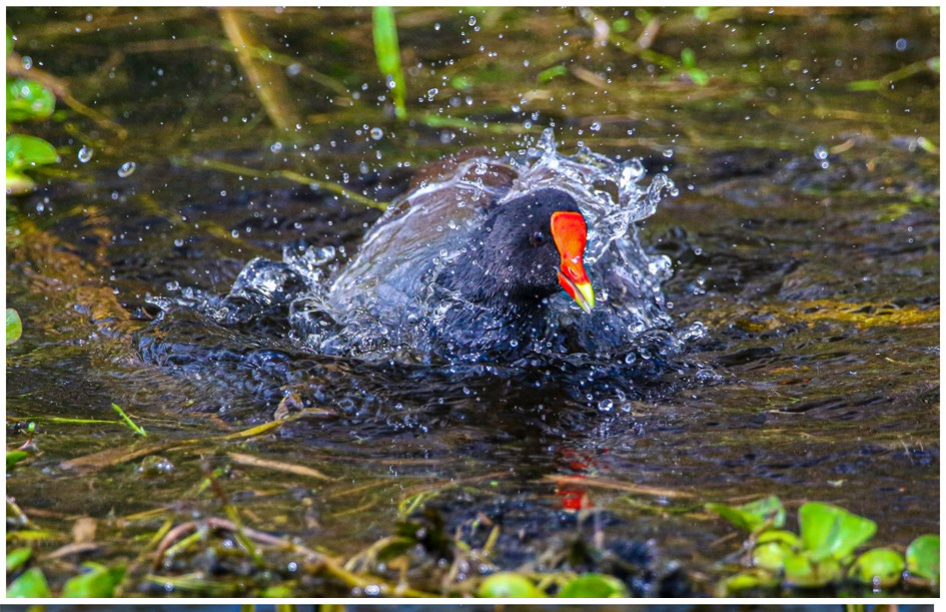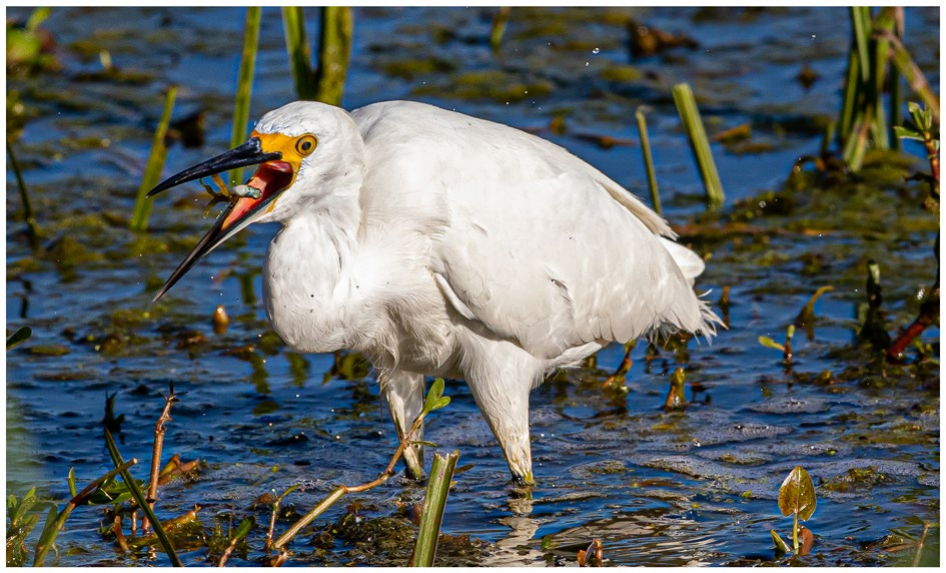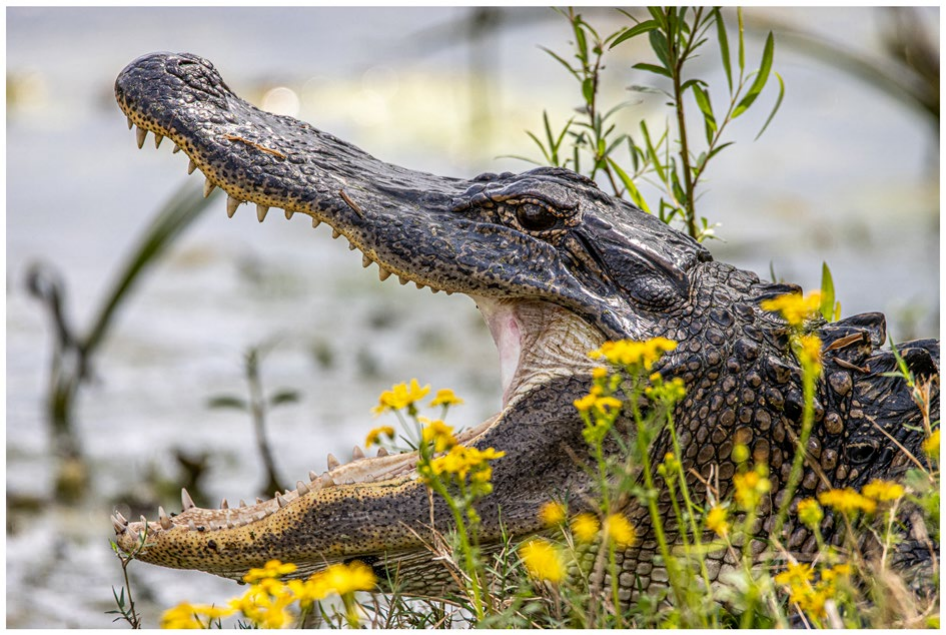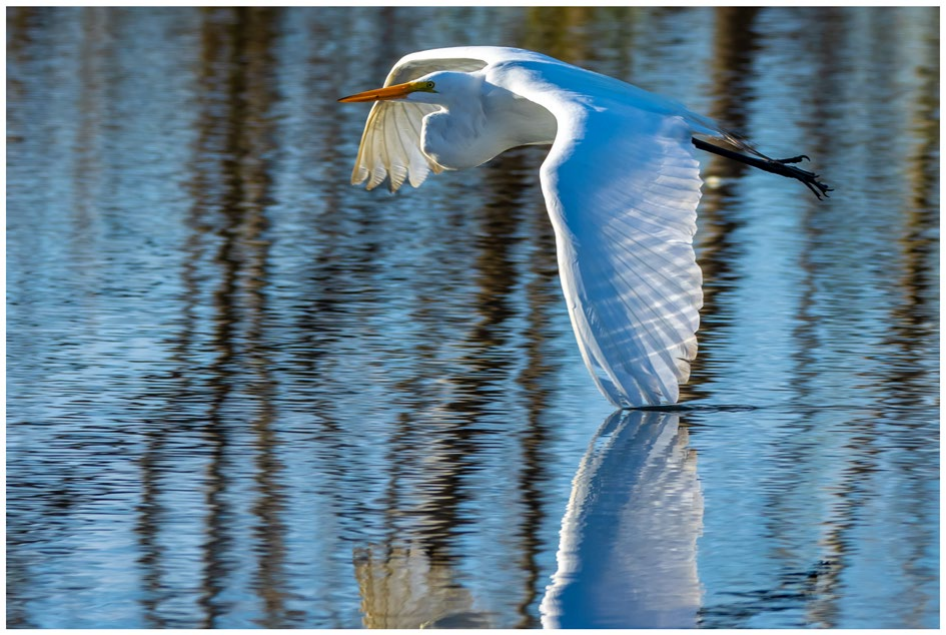# BJUI Compass in the COVID era

**DOI:** 10.1002/bco2.19

**Published:** 2020-05-28

**Authors:** John W. Davis

**Affiliations:** ^1^ BJUI Compass

As we launched the *BJUI Compass* with the March, 2020 inaugural issue, we now know that this was our one and only issue to come out in the pre‐COVID era for most of the world. While my editorial image depicted me wandering the desert in search of quality papers throughout the world, we have all since been shut down from most forms of travel. Meeting after meeting—external and internal—have been cancelled in lieu of virtual meetings. While many of us were already familiar with using Twitter, Facebook, and other social medial platforms to communicate on both professional and personal topics, most of us have now added Zoom, Webex, and other video conferencing platforms to our lives. It is unclear how long it will take for the Covid crisis to pass, but when it does, many have commented that virtual communications will be here to stay in one form or another. As an example, as the flagship *BJUI* journal undergoes its transition from the editorial team of Prokar Dasgupta to Freddie Hamdy, a question came up in internal emails about how to efficiently assign reviewers for papers, and optimize use of the ScholarOne website. Rather than trading multiple long emails on the topic, we quickly setup a Zoom call between myself, Prof Hamdy, and office staff. I could see everyone, talk for a bit, and then share my screen and slides on the topic I have used for lectures in the past. And in a matter of an hour—topic resolved, and we can move on.

Continuing with the Covid crisis theme, for the May 2020 issue, we begin with a novel *review topic* and format organized by Badar Mian from Albany, New York. Dr. Mian is active on Twitter and was part of several posts and conversations about how to manage Covid‐related decisions on patient care in an era when virtually nothing was published on the topic. Twitter became a sort of real‐time bulletin board for information on how to triage and delay services, safety in the OR, and progress with testing. Dr. Mian’s co‐authors continue the topic with some international experiences as narrated by Dr. Fernando Kim, and a literature review by Dr. Paras Shah. The team constructed several useful tables on how to triage surgical urgency—one of the most common decisions many of us have faced in the past several weeks.

Continuing with our normal Compass sections:

- *Clinical utility.* Greer et al from the University of Miami present a highly useful study on reducing post‐operative opioid use for men having scrotal surgery. Their key findings were that they could reduce the opioid prescriptions by about half and use other methods such as NSAIDS and ice as pain relief adjuncts. This is a very timely concept as opioid addiction is such a global threat to our populations, and surgeons can unexpectedly play a role but prescribing way too many opioids at discharge—many of which will sit around unused and/or facilitate future abuse. One of my other career roles is as the Patient Safety and Quality Officer for our urology department at MD Anderson Cancer Center. Our division of surgery last year tackled several projects as a group to improve quality outcomes, and I would put this one up their as one of the top three. I subsequently have cut opioids in half after robotic surgery. Electronic records systems clearly help, as if we have an outlier case that has more pain than usual and needs a refill, we can prescribe within the EMR with a duo‐connect security measure, rather than the old method of requiring paper scripts—this latter point likely a cause of why many providers would over‐prescribe pain meds “just in case.” Of interest, the other two high impact quality projects were DVT prophylaxis, and diabetic screening/post‐op care. Perhaps we can see future papers on these topics.
- *Academic progress*. The paper by Renzulli et al, is a pharmico‐kinetic study in androgen deprivation methods. The introduction is an important review summary, as the field of ADT has changed over the past years to recognize that castrate T levels should really be <20 ng/dL rather than <50 ng/mL. In addition, levels need to be consistently low and absent of micro‐surges or end of dose flares. This study looks specifically at the ATRIGEL delivery system and any effects of age or body weight on its performance. The key conclusion is that this product is effective at maintaining T suppression <= 20 ng/mL and does not need adjustment for weight or age.
- *Innovation*. The paper by Ho et al is an interesting product evaluation with outcomes. Urologists working at multidisciplinary cancer programs will no doubt have a number of consult requests for hydronephrosis from GU and non‐GU malignancies causing extrinsic compression. This metallic ureteric stent has some efficacy in maintaining patency and avoiding nephrostomy—overall 85% success and 33% delayed need for nephrostomy. The authors then present risk factors for which patients would be predicted to fail this method more than others.

Thank you for reading our new journal and I hope more and more authors will consider submissions. Be sure to check if your institution has open access publishing funding or agreements. As we noted at the inaugural issue—open access is open for the whole word of digital access and reading.

Stay Safe.

**Figures:**

“Getting to know your own back yard.”

After last month’s picture of me wandering the desert in Abu Dhabi, I have been on lock down like many/most of you. So for a photo shoot I have to look to my own area. Of interest, just an hour drive to just beyond the Houston city limits is a state park called Brazos Bend State Park. It is a swampy like area with a native alligator population that does not seem to mind or bother the visitors—but social distancing does take on a new meaning here. Bird life is abundant—and for a photo enthusiast an opportunity for that magical “bird‐in‐flight” shot that is a popular challenge for equipment, timing, and patience.

I hope you are all staying safe—perhaps you are also getting to know your local area better while on lockdown.